# The diagnostic accuracy of truncated cardiovascular MR protocols for detecting non-ischemic cardiomyopathies

**DOI:** 10.1007/s10554-021-02462-2

**Published:** 2021-11-09

**Authors:** K. Hirschberg, Sz. M. Braun, O. Paul, M. Ochs, J. Riffel, F. Andre, J. Salatzki, J. Lebel, J. Luu, E. Hillier, M. Finster, H. Vago, B. Merkely, H. A. Katus, M. G. Friedrich

**Affiliations:** 1 Heart and Vascular Center, Semmelweis University, Városmajor utca 68, Budapest, 1122 Hungary; 2 Department of Cardiology, Angiology and Pneumonology, University Hospital Heidelberg, Heidelberg, Germany; 3 Departments of Medicine and Diagnostic Radiology, McGill University Health Centre, Montreal, Canada

**Keywords:** Cardiac imaging techniques, Magnetic resonance, Contrast agent, Cardiomyopathy, T1 mapping

## Abstract

Cardiovascular magnetic resonance imaging is one of the most important diagnostic modalities in the evaluation of cardiomyopathies. However, significant limitations are the complex and time-consuming workflows and the need of contrast agents. The aim of this multi-center retrospective study was to assess workflows and diagnostic value of a short, contrast agent-free cardiac magnetic resonance protocol. 160 patients from Heidelberg, Germany and 119 patients from Montreal, Canada with suspected cardiomyopathy and 20 healthy volunteers have been enrolled. Scans were performed at a 1.5Tesla or 3Tesla scanner in Heidelberg and at a 3Tesla scanner in Montreal. We used single-slice T1 map only. A stepwise analysis of images has been performed. The possible differential diagnosis after each step has been defined. T1-values and color-encoded T1 maps significantly contributed to the differential diagnosis in 54% of the cases (161/299); the final diagnosis has been done without late gadolinium enhancement images in 83% of healthy individuals, in 99% of patients with dilated cardiomyopathy, in 93% of amyloidosis patients, in 94% of patients with hypertrophic cardiomyopathy and in 85% of patients with hypertensive heart disease, respectively. Comparing the scan time with (48 ± 7 min) vs. without contrast agent (23 ± 5 min), significant time saving could be reached by the short protocol. Subgroup analysis showed the most additional diagnostic value of T1 maps in amyloidosis and hypertrophic cardiomyopathy or in confirmation of normal findings. In patients with unclear left ventricular hypertrophy, a short, non-contrast protocol can be used for diagnostic decision-making, if the quality of the T1 map is diagnostic, even if only one slice is available.

## Introduction

Cardiovascular magnetic resonance (CMR) imaging is the reference modality for assessing cardiac morphology and function, with a robust image quality, high accuracy, and excellent reproducibility [1, 2]. Furthermore, CMR has a unique capability of myocardial tissue characterization, which is essential for identifying the presence, extent, severity, and etiology of myocardial injury in diseases such as myocarditis, acute or chronic myocardial infarction, myocardial storage disease, cardiomyopathies [3–7]. Next to edema-sensitive CMR imaging using so-called T2-weighted sequences in the diagnostic workup of left ventricular dilatation, hypertrophy, or dysfunction, CMR can apply contrast-enhanced imaging for identifying myocardial infiltration or scar. The most frequently used technique is called late gadolinium enhancement (LGE) imaging and allows for visualizing infiltration and irreversible injury (necrosis, scar). The observed regional distribution often helps establishing a definitive diagnosis with an impact on patient management [8, 9]. In many clinical scenarios, the early implementation of CMR can save unnecessary diagnostic tests, speed-up decision making and reduce costs. As a significant limitation however, the current workflows for scanning and evaluation of CMR are complex and time-consuming, limiting its practical clinical utility (expensive hardware and software, operating costs, demanding training requirements for staff). Addressing some of these issues would lead to a wider availability for patients [10–16] and help improving informed therapeutic decision-making. Specifically, the need for injecting a contrast agent for tissue characterization in several clinical indications for CMR affects patient safety, comfort, and cost. Gadolinium-enhanced images prolong scan time significantly and require the availability of a physician in case of the rare event of an allergic reaction. Furthermore, some patients have contraindications to gadolinium such as severe kidney dysfunction or a known gadolinium allergy, or patients decline receiving contrast agents due to safety concerns [17, 18]. For these reasons, contrast-agent free protocols, if without loss of relevant information, would be very beneficial.

Over the recent years, the techniques for myocardial T1- and T2-mapping have led to the development of contrast-agent free CMR protocols in patients with suspected cardiomyopathies [19–21]. The measurement of absolute values of global and segmental myocardial T1, displayed in color-encoded T1 maps is a viable alternative to conventional late gadolinium enhancement techniques in the detection of diffuse or focal fibrosis in different cardiac pathologies. Of note, conventional LGE imaging relies on a proper selection of MR sequence settings in order to “null” the signal from normal myocardium. In diffuse myocardial infiltration, especially at earlier stage, this may not be detected on late gadolinium enhanced images [19].

The aim of this multi-center retrospective study was to assess workflows and diagnostic value of a short, contrast agent-free CMR protocol in the evaluation of suspected cardiomyopathies when compared to standard CMR imaging. We aimed at evaluating both, global and regional myocardial abnormalities.

## Methods

### Patients

We performed a retrospective study conducted in the University Hospital Heidelberg, Germany and the McGill University Health Centre, Montréal, Canada. Both centers support large CMR clinical service (> 1000 patients a year). Approval for analysis of imaging data was granted by the local research ethics committees. We selected all patients with suspected cardiomyopathies who had undergone a complete CMR including cine images, T1 mapping and LGE (late gadolinium enhancement) between January 2016 and October 2017 (n = 299). The two cohorts consisted of 160 patients from Heidelberg and 119 patients from Montréal. In addition, 20 volunteers served as a control group for T1 mapping (n = 10 at 1.5T and n = 10 at 3T). All of them were healthy with no significant medical history, no evidence of cardiovascular disease and without any regular medication. In both centers, all patients had provided written informed consent for the CMR examination including the administration of intravenous contrast agent. The patients’ records were de-identified prior to analysis.

### CMR protocol

The CMR procedures were performed by using a 1.5T scanner (Philips Achieva 1.5T CX) and a 3T scanner (Philips Ingenia 3T) in Heidelberg and a 3T scanner (Siemens Magnetom Skyra 3T) in Montréal. 32-element cardiac receiver coils were used for all scanners. Subjects were scanned in supine position with vector ECG-gating. Short and long axis cine images covering the whole left ventricle were acquired using a multi-slice balanced steady state free precession (bSSFP) sequence. T1-mapping was performed using a 2D MOLLI 5s(3s)3s sequence in a single, mid-ventricular short axis slice during breath-hold in end-expiration at late diastole. This sequence is virtually independent from heart rate, but the recorded number of raw images is influenced by the heart rate and a minimum of 8 raw images were acquired with increasing inversion times (TI: 100–5000 ms) at the papillary muscle level (flip angle: 35°) [22]. The reproducibility of T1-mapping has been evaluated during one of our previous studies, showing a small interobserver variability (mean difference of 8.2 ms, and intraobserver (5.9 ms) variability at 1.5T [23]. Late gadolinium enhancement (LGE) images were acquired starting 10 min after administration of Gadobutol (Gadovist, Schering, Berlin, Germany) 0.14 mmol/ kg body weight (1.5T) or 0.1 mmol/kg body weight (3T) employing a T1-weighted inversion recovery-prepared fast gradient echo sequence with an optimized inversion time. Regions with LGE were verified in at least one other orthogonal plane and in the same plane being obtained as a second image after changing the direction of readout [24].

### Image analysis

All CMR scans were analysed using certified post-processing software (cvi42, version 5.2, Circle Cardiovascular Imaging Inc.) by an experienced cardiologist and certified CMR-specialist, who was blinded to the patients’ diagnosis. Left endocardial and epicardial surfaces were contoured manually on the short axis cine images in order to obtain the morphological and functional indices: LV end systolic volume (LVESV), LV end diastolic volume (LVEDV), LV septal thickness, LV ejection fraction (LVEF) calculated from the end systolic and end diastolic volumes. For LV mass calculation, intraventricular septum was included. CMR volume and mass measurements were indexed for body surface area where appropriate. Native T1 maps were generated and analysed by using the same software package. All maps were created by a trained CMR researcher blinded to the original diagnosis and previous results. An R2 cut-off value of 0.9 was used to avoid the generation of false pixels resulting from some form of curve fitting errors. For contouring an offset of 10% was used to avoid partial-volume effects in the subendocardial and subepicardial layers. Global T1 values were calculated as a mean of the 6 segments of the single, mid-ventricular short axis slice. Polar maps were created based on the mid-ventricular short axis slice of the American Heart Association (AHA) 17-segment model [25]. By using our normal reference values calculated from the healthy volunteers, we created specific color-encoded T1 maps to facilitate the visualisation of any pathologic changes. We used green color to highlight areas within normal range of T1 relaxation times, yellow, blue and red colors for increased, decreased T1 values and for blood pool, respectively. Myocardial T1 were considered abnormal if the mean value or at least 10 contiguous pixels (~ 4 mm) had a T1 value out of the normal range. LGE images were visually analysed for the presence or absence of enhancement.

### Three-step blinded analysis of images

All data and images were analysed in three steps to get to a final diagnosis. After each step, the possible number of differential diagnoses were considered. At the first step, only the patient`s history and morphology (cine images) were evaluated. It was followed by the evaluation of the T1 maps (second step). Finally, LGE images were used to further reduce the number of possible differential diagnoses or to verify the suspected diagnosis (third step). After the diagnostic procedure, all patients were assigned to the following groups according to their final diagnosis: normal findings, amyloidosis, DCM (dilated cardiomyopathy), HCM (hypertrophic cardiomyopathy), HHD (hypertensive heart disease), unclear diagnosis, other diagnosis. If no diagnosis could be stated after the third step, no single final diagnosis was given. “Other diagnosis” was stated if any other diagnosis except the above mentioned (normal or amyloidosis or DCM or HCM or HHD) was found.

### Statistical analysis

Categorical data are expressed as percentages and continuous variables as mean ± SD. Statistical significance was defined for p values < 0.05. Groups were compared by using unpaired Student’s t-test. All tests were 2-tailed. Normal reference range for T1 values was calculated from the results of healthy volunteers, defined as mean ± 1.96 SD as previously suggested [19].

## Results

The normal ranges for 1.5T and 3T were 956–1035 ms and 1197–1286 ms, respectively. From the Heidelberg cohort (n = 160), 14 patients were excluded from the final analysis because of an unclear diagnosis or specific ischemic changes or other diagnosis. These patients were assigned to the following groups: Normal, n = 16; amyloidosis, n = 23; DCM, n = 56; HCM, n = 30; HHD, n = 21. From the Montreal cohort (n = 119), 31 patients were excluded because of an unclear diagnosis or other specific diagnoses. These patients were assigned to the following groups: normal, n = 25; amyloidosis, n = 4; DCM, n = 30; HCM, n = 17; HHD, n = 12. After the exclusion of 45 patients, a total of 234 patients were finally analysed.

### Scan time

We analysed the time needed for the CMR examinations. The mean scan time when including post-contrast images was 48 ± 7 min, while images without using contrast-enhanced sequences were acquired in 23 ± 5 min; p < 0.0001.

### Clinical characteristics of patients and CMR results

Baseline clinical characteristics and CMR findings of the healthy volunteers and patients are summarised in Tables 1, 2 and 3. All results of the patients were compared to those of the volunteers.

**Table 1:** Clinical and CMR characteristics of healthy volunteers

	Healthy volunteers, n = 20	
Clinical characteristics		
Age (years)	58.9 ± 11.2	
Gender, n (female/male)	6/14	
BMI (kg/m^2^)	23.6 ± 2.6	
hsTnT (pg/l)	5.8 (3–10), n = 6	
NT-proBNP (µg/ml)	87.8 (20–179), n = 6	
GFR (ml/min/1.73 m^2^ [CKD-EPI])	89.0 ± 11.4	
Creatinine (µmol/l)	86.0 ± 11.8	
Hypertension, n	0	n = 17
Diabetes mellitus, n	0	
Hyperlipidaemia, n	0	
Smoking history, n	0	
Familiy history of CVD	0	
CMR characteristics		
LVEDV (ml/m^2^)	84.5 ± 13.0	
LVESV (ml/m^2^)	32.6 ± 6.1	
LVEF (%)	61.5 ± 3.3	
LV mass (g/m^2^)	48.6 ± 8.2	
Septal thickness (mm)	9.2 ± 1.9	
Presence of LGE/total, n	0/20	
Native T1 value at 1.5T (ms)	995.6 ± 20.2	n = 10
Native T1 value at 3T (ms)	1241.1 ± 22.7	n = 10

Values are expressed as mean ± SD. Number of patients are presented where it differed from the patient group

BMI body mass index, hsTnT high-sensitivity Troponin T, NT-proBPN N-terminal pro b-type natriuretic peptide, GFR glomerular filtration rate, LVEDV left ventricular end-diastolic volume, LVESV left ventricular end-systolic volume, LV left ventricular, LGE late gadolinium enchancement

**Table 2:** Clinical and CMR characteristics of patients from Heidelberg

	Healthy subjects n = 16		Amyloidosis n = 23		DCM n = 56		HCM n = 30		HHD n = 21	
Clinical characteristics										
Age (years)	49.3 ± 18.8		69.0 ± 11.3*		54.3 ± 13.2		55.1 ± 15.9		52.6 ± 8.7	
Gender, n (female/male)	7/9		9/14		24/32		10/20		8/13	
BMI (kg/m^2^)	25.8 ± 3.9		25.4 ± 4.2		26.9 ± 5.1*		27.0 ± 4.6*		31.6 ± 6.2*	
hsTnT (pg/l)	9.0, n = 1		34.8 (11–66)*, n = 9		24.8 (< 3–214), n = 18		9.8 (5–16), n = 5		15.0, n = 1	
NT-proBNP (µg/ml)	< 20.0, n = 1		3001.6 (997–7059)*, n = 9		3119.0 (62–15,089), n = 9		204.0 (24–431), n = 5		408.0, n = 1	
GFR (ml/min/1.73 m^2^ [CKD-EPI])	93.5 ± 16.8		67.9 ± 15.8*		85.5 ± 17.3		86.9 ± 22.9		89.2 ± 17.2	
Creatinine (µmol/l)	86.1 ± 17.0		103.6 ± 23.8*		91.5 ± 20.5		91.7 ± 25.9		91.7 ± 26.4	
Hypertension, n	4	n = 12	6	n = 12	19	n = 43	12	n = 19	20	n = 21
Diabetes mellitus, n	2		2		1		0		3	
Hyperlipidaemia, n	3		6		11		3		3	
Smoking history, n	4		1		13		4		7	
Familiy history of CVD	3		2		12		5		3	
CMR characteristics										
LVEDV (ml/m^2^)	77.5 ± 14.9		82.2 ± 17.7		119.9 ± 37.9*		77.0 ± 18.7		80.4 ± 20.0	
LVESV (ml/m^2^)	30.6 ± 7.5		40.7 ± 16.2*		74.0 ± 36.0*		26.7 ± 11.5*		30.5 ± 10.3	
LVEF (%)	60.8 ± 4.2		51.4 ± 11.7*		40.3 ± 10.3*		66.3 ± 7.7*		62.4 ± 6.2	
LV mass (g/m^2^)	50.4 ± 12.1		93.2 ± 20.5*		69.5 ± 19.9*		78.0 ± 21.6*		70.2 ± 19.4*	
Septal thickness (mm)	9.4 ± 1.6		18.0 ± 2.6*		9.6 ± 1.6		17.8 ± 3.6*		12.9 ± 1.6*	
Native T1 value at 1.5T (ms)	1,011.1 ± 37.9	n = 8	1,164.4 ± 51.5*	n = 14	1,025.6 ± 35.6*	n = 23	1,027.4 ± 39.4*	n = 11	1,010.0 ± 31.1	n = 2
Native T1 value at 3T (ms)	1,239.1 ± 43.3	n = 8	1,407.3 ± 26.6*	n = 9	1,274.6 ± 30.2*	n = 33	1,270.3 ± 32.2*	n = 19	1,247.8 ± 35.8	n = 19

Values are expressed as mean ± SD. Number of patients are presented where it differed from the patient group

BMI body mass index, hsTnT high-sensitivity Troponin T, NT-proBPN N-terminal pro b-type natriuretic peptide, GFR glomerular filtration rate, LVEDV left ventricular end-diastolic volume, LVESV left ventricular end-systolic volume, LV left ventricular, LGE late gadolinium enchancement

*p < 0.05 when compared to healthy volunteers from Table 1

**Table 3:** Clinical and CMR characteristics of patients from Montréal

	Normal finding, n = 25		Amyloidosis, n = 4		DCM, n = 30		HCM, n = 17		HHD, n = 12	
Clinical characteristics										
Age (years)	49.7 ± 18.2		72.3 ± 17.9		57.4 ± 12.3		55.4 ± 11.5		60.0 ± 13.0	
Gender, n (female/male)	9/16		2/2		9/21		6/11		3/9	
BMI (kg/m^2^)	26.9 ± 5.6*		23.9 ± 4.0		28.3 ± 5.1*		28.4 ± 4.5*		29.4 ± 6.3*	
NT-proBNP (µg/ml)	N/A		753.0, n = 1		321.6 (36–858)*, n = 11		198.5 (22–375), n = 2		120.0, n = 1	
Creatinine (µmol/l)	76.2 ± 15.13, n = 15		109.7 ± 46.3, n = 3		92.6 ± 22.4, n = 22		82.2 ± 10.6, n = 11		88.4 ± 24.1, n = 5	
Hypertension, n	6	n = 17	1	n = 3	9	n = 25	5	n = 9	6	n = 6
Diabetes mellitus, n	1		1		2		2		2	
Hyperlipidaemia, n	5		2		4		5		3	
Smoking history, n	1		0		7		0		0	
Familiy history of CVD	4		0		3		3		1	
CMR characteristics										
LVEDV (ml/m^2^)	69.7 ± 18.0*		65.2 ± 36.79*		132.8 ± 39.6*		64.2 ± 16.4*		68.0 ± 20.3*	
LVESV (ml/m^2^)	25.1 ± 10.0*		17.5 ± 17.73		92.3 ± 34.6*		16.7 ± 9.2*		31.1 ± 17.1	
LVEF (%)	64.5 ± 7.5		46.0 ± 14.0*		31.2 ± 9.4*		74.5 ± 10.2*		56.3 ± 13.1	
LV mass (g/m^2^)	63.1 ± 12.6*		131.8 ± 32.7*		103.7 ± 29.8*		103.1 ± 29.2*		88.7 ± 35.2*	
septal thickness (mm)	8.8 ± 3.1		14.7 ± 2.1*		8.9 ± 2.1		13.6 ± 3.3*		11.2 ± 1.8*	
Native T1 value at 3T (ms)	1,241.0 ± 29.7		1,439.5 ± 71.1*		1,306.1 ± 34.4*		1,292.2 ± 34.5*		1,300.8 ± 32.7*	

*p < 0.05 when compared to healthy volunteers from Table 1

### Patients with normal findings

In both cohorts, T1 values did not differ from that of the healthy volunteers (995.6 ms at 1.5T and 1241.1 ms at 3T), confirming the absence of pathological alterations of the myocardium.

**Fig. 1:**
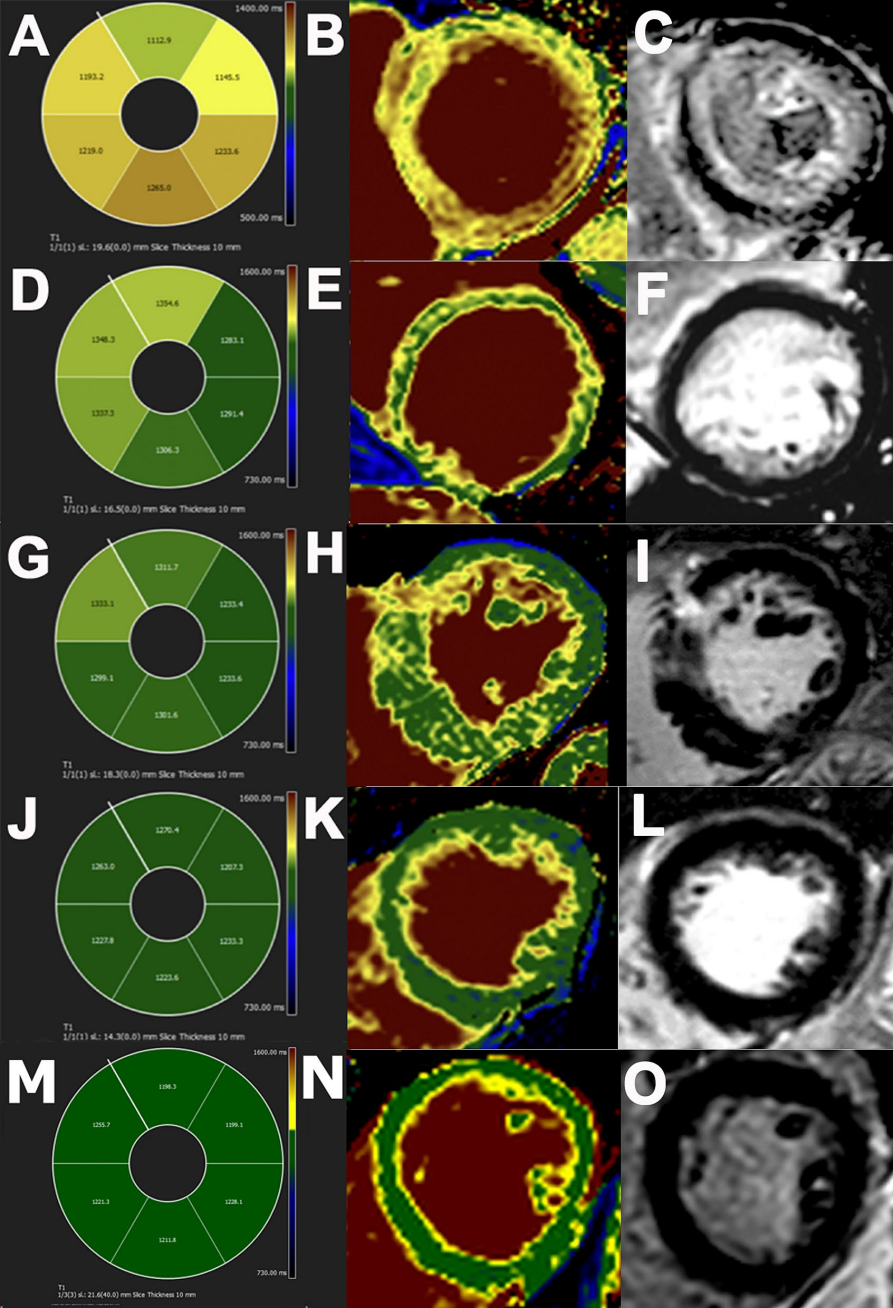
**A–C** T1 polar map, color-coded T1 map and the correspondent LGE image of a patient with amyloidosis. **D–F** T1 polar map, color-coded T1 map and the correspondent LGE image of a patient with DCM. **G–I** T1 polar map, color-coded T1 map and the correspondent LGE image of a patient with HCM. **J–L** T1 polar map, color-coded T1 map and the correspondent LGE image of a patient with hypertensive heart disease (HHD). **M–O** T1 polar map, color-coded T1 map and the correspondent LGE image of healthy volunteer

Figure 1M–O shows a representative color-encoded T1 map in a healthy subject.

### Amyloidosis

As expected, LV mass indices and septal thickness showed a robust increase in both cohorts (93.2 g/m^2^ and 131.8 g/m^2^) when compared to the healthy volunteers (48.6 g/m^2^, p < 0.05). T1 was also significantly increased; 1164 ms at 1.5T and 1407 ms and 1439 ms at 3T (Tables 2 and 3, Fig. 1A–C).

### Dilated cardiomyopathy

LVEDV (119.9 ml/m^2^ and 132.8 ml/m^2^), LVESV (74.0 ml/m^2^ and 92.3 ml/m^2^), LV mass indices (69.5 g/m^2^ and 103.7 g/m^2^) were significantly higher and LVEF (40.3% and 31.2%) was significantly lower in both cohorts as compared to the healthy volunteers (LVEDV 84.5 ml/m^2^, LVESV 32.6 ml/m^2^, LV mass 48.6 g/m^2^, LVEF 61.5%, p < 0.05, respectively).

T1 values were slightly, but significantly elevated; 1025.6 ms at 1.5T and 1274.6 ms and 1306.1 ms at 3T (Tables 2 and 3, Fig. 1D–F).

### Hypertrophic cardiomyopathy

The difference between the T1 values was not as prominent as in amyloidosis, albeit still significantly higher than normal; 1027.4 ms at 1.5T and 1270.3 ms and 1292.2 ms at 3T vs. 995.6 ms at 1.5T and 1241.1 ms at 3T, respectively (p < 0.05). The most prominent differences in both groups were in the LV mass indices (78.0 g/m^2^ and 103.1 g/m^2^ vs. 48.6 g/m^2^, p < 0.05) and septal thickness (17.8 mm and 13.6 mm vs. 9.2 mm, p < 0.05). LVESV decreased significantly (Tables 2 and 3, Fig. 1G–I).

### Hypertensive heart disease

There were 33 patients diagnosed with HHD in the two cohorts together. BMI was significantly higher in both cohorts (31.6 kg/m^2^ and 29.4 kg/m^2^ vs. 23.6 kg/m^2^, p < 0.05). Both LV mass indices and septal thickness were significantly higher in both the Heidelberg and Montreal cohorts (70.2 g/m^2^ and 88.7 g/m^2^ vs. 48.6 g/m^2^; 12.9 mm and 11.2 mm vs. 9.2 mm, respectively, p < 0.05). In the Heidelberg cohort, there was no significant difference between the T1 values (1248 ms vs. 1239 ms at 3T and 1010 ms vs. 1011 ms at 1.5T, both p > 0.4). In the Montreal cohort, T1 values were significantly higher (1301 ms; p < 0.05). (Tables 2 and 3, Fig. 1J–L).

### T1-mapping in the stepwise diagnostic approach

The evaluation of the patients’ data and images was performed in three steps by an experienced cardiologist, blinded to the diagnoses as stated on the clinical CMR report. Table 4 shows the reduction of differential diagnosis under consideration when adding T1 mapping to the analysis.

**Table 4:** Results of the three-step evaluation

Final diagnosis	Total number of patients	Correct diagnosis (history + morphology)	Correct diagnosis (history + morphology + T1-mapping)	Correct diagnosis (history + morphology + T1-mapping + LGE)
Healthy subjects	41	8 (19.5%)	34 (83%)	41 (100%)
Amyloidosis	27	0 (0%)	25 (93%)	27 (100%)
DCM	86	64 (74%)	85 (99%)	86 (100%)
HCM	47	16 (34%)	44 (94%)	47 (100%)
HHD	33	6 (18%)	28 (85%)	33 (100%)

The percentage of hitting the final diagnosis made by the inclusion of LGE images is shown at each diagnostic steps

Values show the total number of correct diagnoses made after each step

LGE late gadolinium enhancement, DCM dilated cardiomyopathy, HCM hypertrophic cardiomyopathy, HHD hypertensive heart disease

### Step 1

After checking the patient`s history and completing the morphological analysis of 234 patients, in 94 cases (40%) a final diagnosis could be made (74% of DCM cases, 34% of HCM cases, but none of the amyloidosis cases could be diagnosed at this step).

### Step 2

In this step, polar maps and color-coded T1 maps were added to the cine images. In additional 122 cases a diagnosis could be established (216 from 234 cases, 92% in total). The greatest benefit of T1-mapping was seen during the evaluation of patients with amyloidosis, followed by HCM. In amyloidosis patients, a globally high native myocardial T1 reliably visualized diffuse myocardial infiltration. T1-mapping was sufficient for the diagnosis in 25 cases, representing 93% of all amyloidosis cases (Table 4). In HCM patients, focal fibrosis at the RV insertion points was often well visualised on native color-coded T1 maps. T1-mapping helped to diagnose 28 additional cases of HCM (60% of all HCM) and 22 more cases of HHD (67% of all HHD). Diagnostic step 1 and 2 together made it possible to diagnose 44 cases of HCM (94% of all HCM) and 28 cases of HHD (85% of all HHD). Looking at the subgroup of patients with normal finding, T1-mapping ruled out the possibility of myocardial disease in 26 more cases (63% of all individuals in this group), which means 34 cases together with the first step (83% of all individuals in this group).

### Step 3

**Fig. 2:**
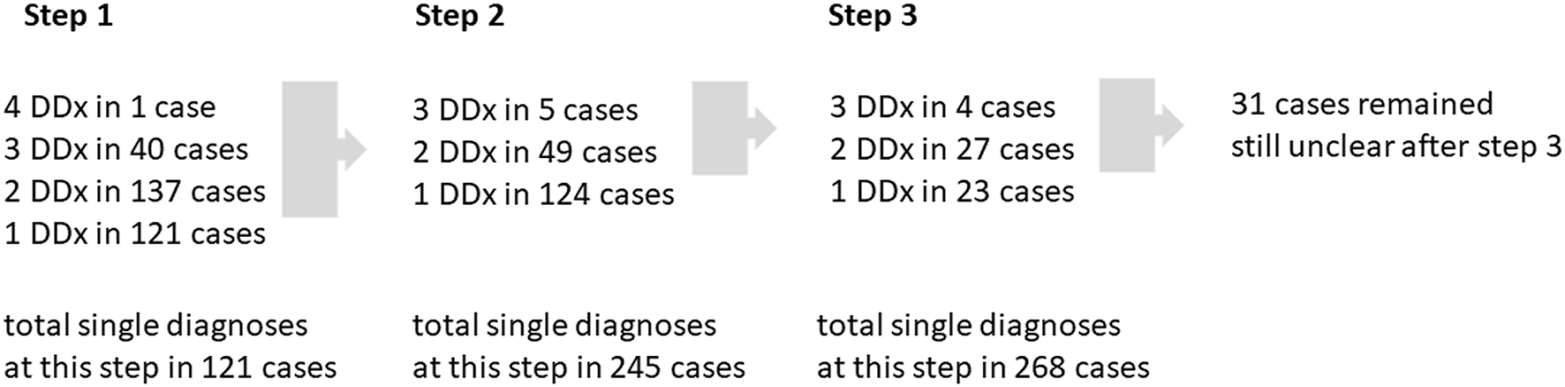
Narrowing of differential diagnoses (DDx) at each step

The final step of the evaluation included the additional analysis of LGE images. There were 31 cases, where neither LGE nor the previous methods were able to reduce the possible number of differential diagnoses to a single one. Just like the patients with “other diagnoses" (n = 14), these patients were also excluded from the final interpretation. For the correct final diagnosis, LGE was essential in 18 cases (8%). Figure 2 shows the stepwise narrowing of differential diagnoses (DDx) after each step.

**Fig. 3:**
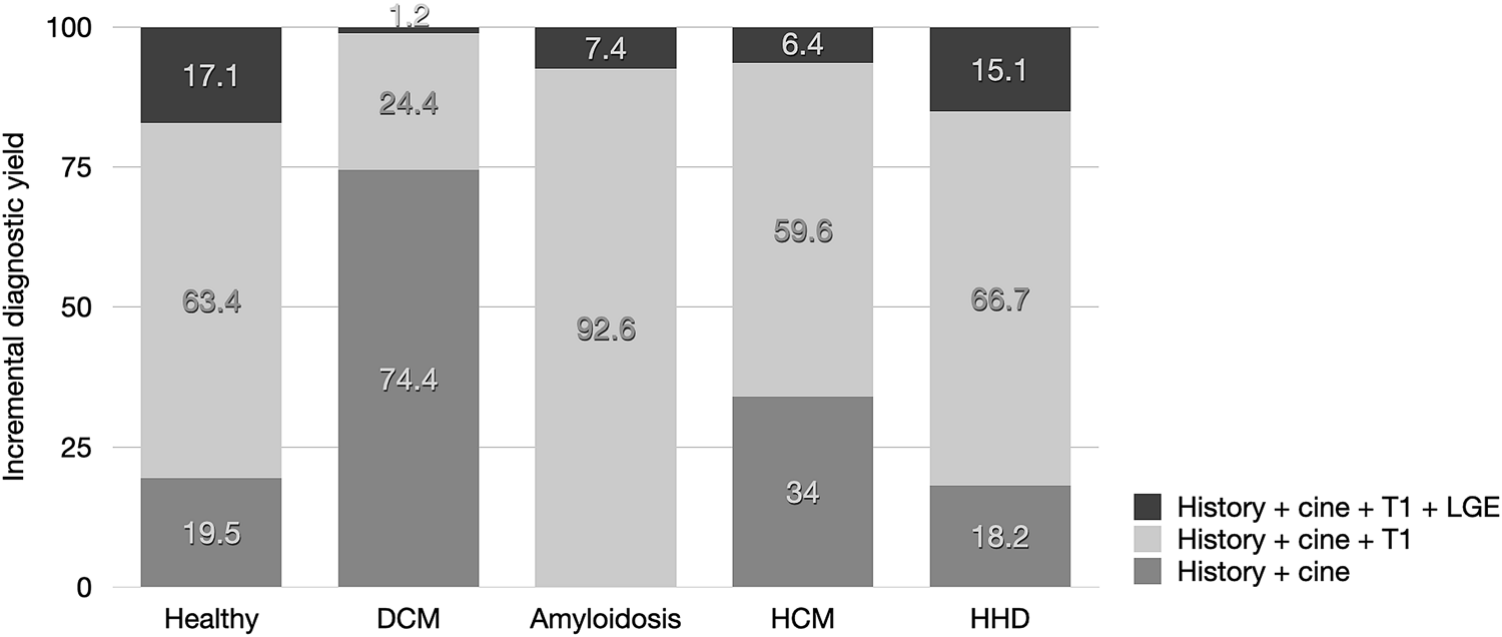
Incremental diagnostic yield in healthy individuals and in DCM, amyloidosis, HCM and HHD patients

Representative examples of color-encoded T1 maps from a healthy volunteer and from each subgroup are shown in Fig. 1. The incremental diagnostic yield is shown in Fig. 3.

## Discussion

Our study in two experienced CMR centres showed that in 92% of patients with suspected non-ischemic cardiomyopathies, scan time and associated costs could be significantly reduced by omitting contrast-enhanced images without impairment of diagnostic performance. In the remaining 8% only, readers required contrast-enhanced LGE images were necessary for diagnostic decision-making. The results however vary between cardiomyopathies. While the performance of non-contrast enhanced protocols was excellent in DCM (99%), in HCM (94%) and amyloidosis (93%), the accuracy was below 90% in patients with hypertensive heart disease. In 83% of normal individuals, readers felt comfortable ruling out cardiac disease without LGE images.

Our findings have several clinical implications: Contrast agents may be omitted in HCM and DCM, the two most frequent cardiomyopathies. The results do also provide reassurance that in patients with suspected cardiomyopathies in whom the injection of gadolinium is contraindicated, a CMR with a high diagnostic yield can be performed even without the use of a contrast agent.

Our results also support a non-rigid protocol, i.e. advancing to contrast-enhanced images only in the few cases where the diagnosis cannot be established based on cine and mapping alone. Such a strategy would save costs, albeit would require a knowledge-based decision during the scan. One could even consider offering patients a shortened, non-contrast protocol and add the contrast-enhanced part at a second scan, if necessary.

T1 maps with diagnostic quality are an essential component in this approach. The quality and utility of T1 mapping requires suitable hardware and software, as well as expertise and experience of the readers. Furthermore, the quality of T1 maps may vary, and the accuracy also relies on local quality standards such as center-specific normal values and validation in phantoms. As for LGE imaging, T1 mapping should ideally be applied as a stack as an incomplete coverage of the LV reduces its sensitivity for focal lesions (e.g. apical type of hypertrophic cardiomyopathy). In addition, while the global T1 values in our healthy subjects were all within these reference ranges, in some few cases, mild, clinically not relevant, focal changes might have occurred [26].

We observed a very consistent, strong T1 increase in amyloidosis, confirming the high diagnostic accuracy of T1-mapping in cardiac amyloidosis [6, 19, 27]. Considering the difficulties of LGE imaging in these patients [28], our results support CMR protocols without contrast agents in these patients.

Our findings of a globally increased T1 in DCM are consistent with previous reports [29], including its co-location with LGE [30, 31]. The diagnosis of DCM is based on the presence of reduced myocardial function and volume dilatation in the absence of ischaemic heart disease [32]. The importance of morphological and functional analysis can be clearly seen in our three-step diagnostic protocol, where 74% of DCM patients have been diagnosed based on morphology alone and 99% when adding mapping. In clinical scenarios, it can be challenging to diagnose the disease at early stages. For such cases, T1-mapping may be very useful especially for early anti-fibrotic medication [32].

In patients with HCM, we found significantly higher myocardial T1 values when compared to healthy volunteers. The focal elevation of T1 times, mostly at the insertion zones of the right ventricle, were corresponding with abnormal LGE in these regions [33]. Furthermore, in 15% of HCM cases, T1-mapping revealed more focal lesions than did contrast-enhanced LGE imaging. This observation is consistent with previous reports on an additional value of T1 mapping beyond LGE imaging [34]. We encountered 3 cases with negative T1 maps, likely because the incomplete coverage of the left ventricle by just one mid-ventricular slice. It is known, that T1 may very between different regions of the left ventricle, a strong argument for better coverage by more slices [35].

In arterial hypertension, myocardium remodeling occurs due to cardiomyocyte hypertrophy, fibroblast stimulation and increased collagen deposition. Both, left ventricular hypertrophy and the accumulation of interstitial collagen fibers are progressive [36]. The progressivity of remodeling can be the explanation to the difference of T1 values in our patient cohorts. Patients from Montréal showed a distinct T1 time elongation, while patients from Heidelberg had only a slightly increased mean T1 value, which suggests that the patients from Heidelberg were at an early stage of the disease. In the HHD subgroup, 76% of patients showed a positive T1 map in contrast with the 33% with LGE imaging. Although T1 mapping patterns are not always disease-specific, T1-mapping has a clear advantage in revealing early fibrotic transformation.

In our study, a major contribution of T1-mapping was the addition of diagnostic information in patients with left ventricular hypertrophy of unknown origin. The absolute value of T1 and its regional distribution pattern help differentiate between HCM, amyloidosis and hypertensive heart disease [19, 37, 38].

Nadjiri et al. recently evaluated a shortened protocol in a clinical setting in 160 patients [39]. The authors investigated a more heterogenous patient collective compared to our patients, since we focused only on the evaluation of suspected cardiomyopathies and acute cardiac pathologies such as myocardial infarction or myocarditis has been excluded. Similar to our results, Nadjiri et al. showed that a shortened protocol comprised only of myocardial T1 mapping and cine images can discriminate patients who will benefit from a full contrast CMR protocol from those who do not.

T1 mapping, especially when showing an increase of myocardial T1, covers a wider range of myocardial pathology as compared to T2 mapping [21]. T2 on the other hand can specifically identify acute myocardial injury through visualizing myocardial edema. We omitted T2 mapping in our study because in the setting of chronic myocardial disease, T1 mapping has a broader range of detectable myocardial pathologies.

In summary, the following pragmatic approach for a shortened, contrast agent-free CMR protocol could be clinically applied in patients with suspected cardiomyopathy: (1) cine images and T1 mapping are acquired and immediately analyzed (while patient is in the scanner). A normal myocardial T1 and normal ventricular morphology and function as assessed in a set of diagnostic T1 maps and cine images allow for ruling out cardiomyopathy. (2) In case of LV hypertrophy and typical T1 abnormalities indicating HCM or amyloidosis, no LGE images and thus no contrast application would be necessary to rule in HCM or amyloidosis. In all other cases, the scan could be expanded to a full protocol with contrast-enhanced images or a focused contrast-enhanced CMR scan could be scheduled.

## Limitations

There are several, very important limitations of this study. Besides its retrospective design, our study may have been affected by significant selection bias, since only patients with suspected CMP and a clear final diagnosis of a nonischemic CMP were included. We excluded acute clinical syndromes, ischemic pathologies, and rare diagnoses such as arrhythmogenic cardiomyopathy, cardiac involvement of systemic diseases, sarcoidosis etc. Therefore, our results are not applicable to acute myocardial injury and infrequent cardiomyopathies. We believe would however expect similarly positive results. Furthermore, sensitivity and specificity and AUC could not be calculated from these cohorts. We only analyzed one midventricular short axis T1 map, and T2 maps were not included. Thus, updated protocols as now used in many centres would likely achieve even better results. It is also important to state, that omitting LGE imaging may forfeit the opportunity to acquire additional prognostic information.

## Conclusions

Our results indicate that CMR using a truncated protocol of combined functional (cine) images and a single-slice T1 map may in most of patients be sufficient to rule out or confirm the diagnosis of several non-ischemic cardiomyopathies, specifically hypertrophic and dilated forms. These protocol allow for omitting the administration of contrast agents and could significantly reduce the duration and cost of CMR exams. These findings should be confirmed with updated CMR protocols using multi-slice T1 and T2 maps, and in larger, prospective studies.

## Data Availability

On request from the corresponding author.
